# Transcriptome analysis reveals the pathogenesis of spontaneous tibial dyschondroplasia in broilers

**DOI:** 10.3389/fgene.2024.1434532

**Published:** 2024-07-29

**Authors:** Kai Shi, Yongfu Wu, Xusheng Jiang, Xiangping Liu, Yuesong Du, Chungang Feng, Dongfeng Li

**Affiliations:** ^1^ College of Animal Science and Technology, Nanjing Agricultural University, Nanjing, Jiangsu, China; ^2^ Ningbo Academy of Agricultural Sciences, Ningbo, China

**Keywords:** tibial dyschondroplasia, yellow-feather broilers, transcriptome, bone quality, growth plate

## Abstract

Tibial dyschondroplasia (TD) is a severe bone disease that affects fast-growing broiler chickens and causes economic loss. Despite previous studies, the regulatory mechanism of TD remains unclear and is thought to be primarily based on thiram induction, which may differ from that of naturally occurring diseases. To better understand TD, a digital X-ray machine was used in the present study to determine its incidence in four hundred yellow-feathered broiler chickens. The results showed that the incidence of TD was 22% after 6 weeks and gradually decreased after 8 and 10 weeks. The body weight of broilers with TD decreased significantly compared to that of NTD broilers. In addition, the length and density of the tibia were reduced after eight and 10 weeks, and the density of the tibia was reduced after 6 weeks compared with the NTD chickens. This study also examined tibial quality parameters from TD (n = 12) and NTD broilers (n = 12) and found that bone mineral content, bone mineral density, bone ash content, calcium content, and phosphorus content were significantly reduced in TD broilers. Transcriptome analysis revealed 849 differentially expressed genes (DEGs) in the growth plate between TD (n = 6) and NTD groups (n = 6). These genes were enriched in ECM-receptor interaction, cytokine-cytokine receptor interaction, calcium signaling pathway, and TGF-β signaling. Genes encoding the alpha chain of type XII collagen, that is, *COL1A1*, *COL5A1*, and *COL8A1*) were identified as critical in the regulatory network of TD. Gene set enrichment analysis (GSEA) revealed that the pathways of cartilage development, circulatory system development, and nervous system development were changed in the growth plates of TD birds. In the blood transcriptome, 12 DEGs were found in TD (n = 4) and NTD chickens (n = 4), and GSEA revealed that the pathways from TD broilers’ blood related to the phagosome, linoleic acid metabolism, monoatomic ion homeostasis, and calcium ion transport were downregulated. This study provides a comprehensive understanding of TD, including its effects on tibial quality, tibial changes, and the circulatory system, along with identifying important genes that may lead to the development of TD.

## 1 Introduction

Tibial dyschondroplasia (TD), caused by the death of cartilage cells in the tibial growth plate owing to poor blood supply, is a bone disease that affects young chickens and leads to slow growth, lameness, and non-mineralized growth plates in the legs (Mehmood et al., 2017). Birds affected by TD have lower disease resistance and growth performance and are more susceptible to osteomyelitis (Shahzad et al., 2015). TD is a complex disease that is influenced by a variety of factors, such as heredity, nutrition, and environment. A previous study showed that the incidence of TD in chickens increased after three generations of selection for high TD rates (Riddel, 1976). The relationships among TD, body weight, carcass component weight, and abdominal fat weight have been investigated to develop strategies to reduce the incidence of TD while promoting growth (Zhang et al., 1995). Manganese deficiency has also been found to increase the risk of TD by causing abnormal morphology and irregular arrangement of chondrocytes in the hypertrophic and proliferative zones of the tibial growth plate (Wang et al., 2021). Increased exercise in chickens could improve leg health and reduce the incidence of TD (Kaukonen et al., 2017). In addition, the presence of thiram, a pesticide residue in the diet, has been linked to an increased incidence of TD and has been used to create a TD model (Subapriya et al., 2007; Liu et al., 2021; Liu et al., 2022).

Recent studies have shown that TD is related to cartilage development, blood vessel formation, and cell apoptosis (Farquharson and Jefferies, 2000; Jahejo and Tian, 2021). Thiram-induced TD was found to be related to the cytochrome C/Bax/caspase-3/TSC1/mTOR signaling pathway, which mediates autophagy and chondrocyte apoptosis (Fakhar-E-Alam Kulyar et al., 2022; Quan et al., 2024). Autophagy-mediated mechanism via the TSC1/mTOR signaling pathway was detected in thiram-induced tibial dyschondroplasia of broilers (Nawaz et al., 2023). In addition, the inhibition of BMP/Smad, IHH/PTHrP, and AKT/PI3K signaling pathways and activation of the JAK/STAT signaling pathway have been associated with TD (Yao et al., 2020; Jiang et al., 2024). Numerous studies have been conducted to gain insights into TD using transcriptome sequencing technology, which detects the expression changes of individual genes in different physiological states, and has been widely used in animal breeding to discover essential genes that influence traits (Shao et al., 2022). It was found that 141 mRNA, 10 miRNA, 23 lncRNAs, and 35 circRNAs were differentially expressed in the tibia of TD and NTD chickens, these genes and non-coding RNAs are involved in the MARK, Hippo, and TGF-β signaling pathways (Lu et al., 2022). In a recent study, thiram activity was found to enhance pathological remodeling of the ECM by decreasing glycolysis and activating hexosamine and glucuronic acid signaling pathways. Hyperglycemia is considered an important function in ECM overproduction and a driving force in TD (Mo et al., 2023). Another study identified 293 DEGs in avian angiogenesis that were inhibited after thiram ingestion and differentially expressed genes from blood involved in the interaction between neuroactive ligands and receptors, actin cytoskeleton regulation, and the MAPK signaling pathway (Jahejo et al., 2020). Wu et al. found that the level of lncRNA MSTRG.74.1 was significantly increased and could regulate chicken chondrocyte development through increasing the level of BNIP3 (Wu X. et al., 2024). Others proved that miR-203a could influence chondrocyte development by targeting Runx2 gene (Wu S. et al., 2024), miR-181b-1-3p can relieve the negative effect of thiram on cartilage proliferation and differentiation through activation of Wnt/β-catenin signaling pathway (Sun et al., 2023). Although many studies have investigated the pathogenesis of TD, its regulatory mechanisms remain unknown. To date, research has focused primarily on thiram-induced diseases, which may differ from their exact pathogenesis.

In the present study, we detected the occurrence of TD of yellow-feathered chickens at 6, 8, and 10 weeks based on a radiographic technology that were a reliable method to determine TD in broilers (Pelicia et al., 2012). The tibial growth plate and blood samples were collected at 6 weeks to determine the bone quality parameters and perform transcriptome analysis. Results indicated that TD decreased the bone quality and changed gene expression of tibial growth plates (*COL1A1*, *COL5A1*, and *COL8A1*) and blood (*EPSTI1*, *USP41*, and *SLC5A7*), which could help uncover the underlying mechanisms and identify practical molecular markers for TD.

## 2 Materials and methods

### 2.1 Animals

Four hundred specialized native Chinese, yellow-feathered meat chickens were raised under the same conditions from when they were 1 day old at Jiangsu Lihua Animal Husbandry Co., Ltd. (Changzhou, China). At 6, 8, and 10 weeks of age, individual cockerel were examined for the TD phenotype using a digital X-ray machine. In addition, another four hundred broilers were reared and sacrificed after 12 h of fasting for sampling via jugular phlebotomy at 6 weeks.

### 2.2 Assessment of tibial morphology

The tibial morphology parameters were measured using ImageJ software (v1.8.0.112). During the measurement, a straight line was drawn from the proximal to distal extremities of the tibiotarsus to determine the tibial length. Another straight line was drawn in the middle of the first straight line to calculate the tibial diameter.

### 2.3 Tibial quality parameters detection

After the tibias were thawed, a dual-energy X-ray absorptiometry was used to detect bone mineral density (BMD) and bone mineral content (BMC) through an InAlyzer (MEDIKORS, Korea) according to the manufacturer’s procedure. Breaking strength (BBS), stiffness and Young’s modulus (YM) were measured based on a universal material testing machine (LR10K Plus, Lloyd Instruments Ltd., United Kingdom) according to the three-point bending test.

The bone strength was assessed by collecting all fragments of broken bones to determine the percentage of ash based on the fat-free dry weight. The bones were dehydrated by drying at 105°C for 24 h and then defatted with petroleum ether for 48 h. After extracting the water and fat, the samples were dried at 105°C until a constant weight was reached in an oven (GPL-70, Labotery Instrument Equipment Co., Ltd., Tianjin, China). The ash content was determined after 24 h of incineration at 600°C in an electric furnace (DK-98-II, Taisite Instrument Ltd., Tianjin, China). The bone ash content was expressed as the ratio of ash weight to fat-free dry weight. The contents of calcium and phosphorus in the ash were determined using EDTA complexometric titration and ammonium molybdate spectrophotometric methods (Li et al., 2023).

### 2.4 RNA extraction and transcriptome

Tibial metaphyseal growth plates from TD (n = 6) and NTD broilers (n = 6) and blood samples from TD (n = 4) and NTD chickens (n = 4) at 6 weeks were collected and frozen in liquid nitrogen, these samples were extracted total RNA and performed transcriptome sequencing. RNA extraction and extraction of the tibial metaphyseal growth plates and blood were performed as previously described [9]. The mRNA was isolated using the Ribo-ZeroTM Magnetic Kit (Epicentre, Madison, WI, United States), and the Hieff NGS^®^ Ultima Dual-mode RNA Library Prep Kit (Yeasen, Shanghai, China) was used to prepare the mRNA library. Paired-end transcriptome sequencing was performed using the Illumina sequencing platform (Genedenovo Biotechnology Co., Ltd., Guangzhou, China). The raw data obtained were cleaned of low-quality reads using Fastp software (v0.21.0), Q20, Q30, and GC contents were calculated, and clean reads were generated and aligned with the chicken reference genome (GRCg6a, GCA_000002315.5). Transcripts per kilobase million (TPM) were used to determine gene expression levels. The DESeq2 R package (v1.42.0) was used to detect differentially expressed genes (DEGs). Genes with an adjusted *P*-value <0.05 and |log2 fold change| > 1 were considered significantly changed. Gene Ontology (Go) analysis was performed using the online software DAVID (https://david.ncifcrf.gov/summary.jsp), and Kyoto Encyclopedia of Genes and Genomes (KEGG) pathways analysis was performed using the R package ClusterProfiler (v4.10.0).

### 2.5 Statistical analysis

All data were analyzed using SPSS statistical software (version 25.0), and statistical significance was calculated according to Student’s *t*-tests. Results are shown as mean ± standard deviation. *P* < 0.05 was considered a statistically significant difference.

## 3 Results

### 3.1 Phenotype identification of broilers with TD

**FIGURE 1:**
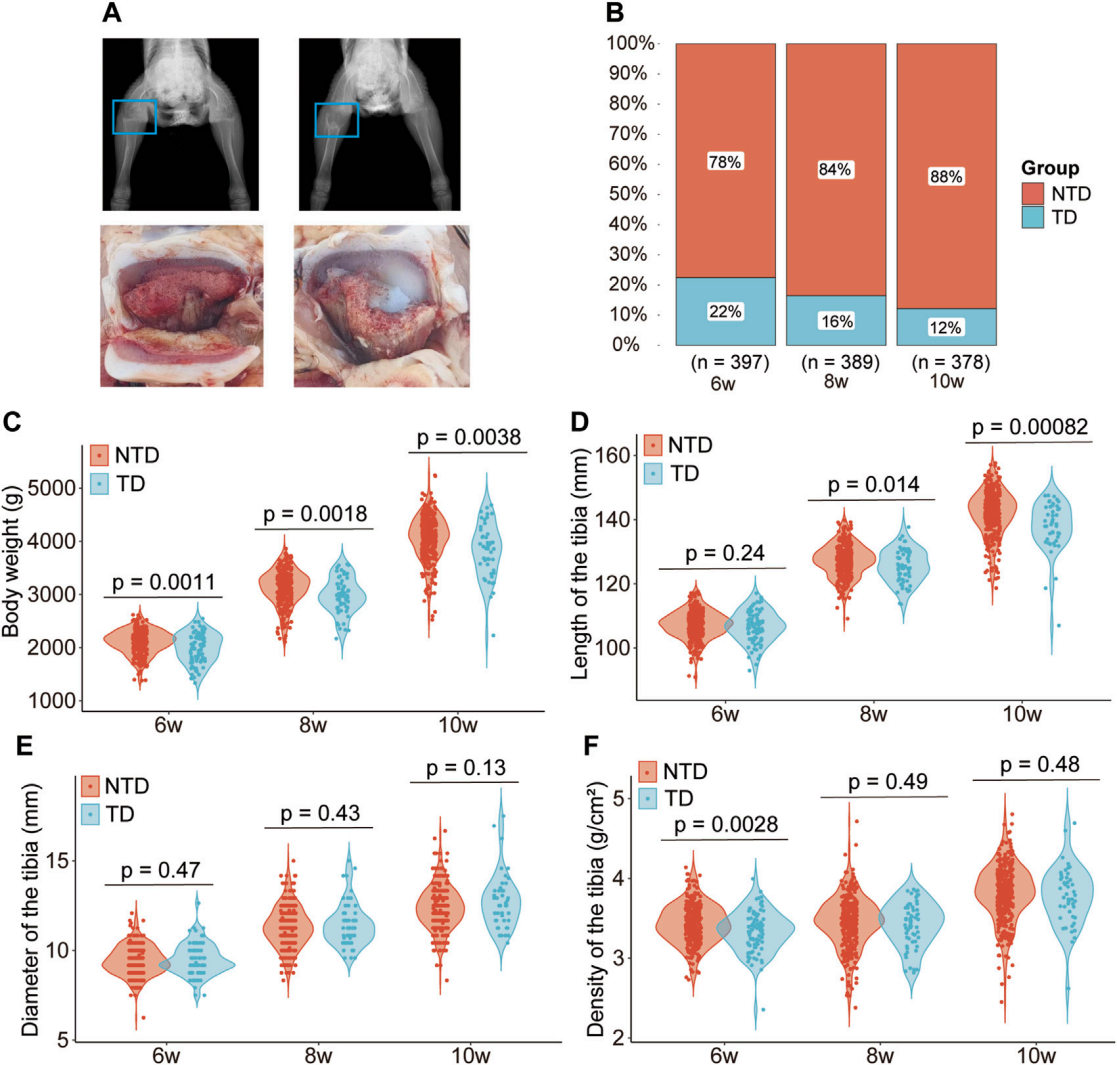
Phenotype differences between Tibial dyschondroplasia and non-tibial dyschondroplasia broilers. Radiograph and anatomical drawing of TD and NTD chickens at 6 weeks of age **(A)**; morbidity of TD at 6, 8, and 10 weeks of age **(B)**; difference in body weight **(C)**, length of the tibia **(D)**, diameter of the tibia **(E)**, and density of the tibia **(F)**.

At 6 weeks of age, the metaphysics of the tibia from TD broilers always showed irregular cartilage, while NTD chickens’ metaphysics of the tibia were smooth based on digital X-ray technology. Avascular and non-mineralized chondrocytes accumulated in the growth plate and formed white jade cartilage plugs in the tibial metaphysis in the TD group. The growth plates of TD broilers showed obvious thickening and reduced blood vessels (Figure 1A). The morbidity of TD in the population was 22, 16, and 12% at 6, 8, and 10 weeks, respectively (Figure 1B). The body weight of broilers in the TD group was significantly lower than that of the NTD group after 6, 8, and 10 weeks (Figure 1C). Tibia length was significantly reduced in TD birds after 8 and 10 weeks compared to that of the NTD group (*p* < 0.05). The tibia diameter showed no significant difference (*p* > 0.05), while the tibial density was noticeably reduced only in the sixth week (*p* < 0.05) (Figures 1D–F).

### 3.2 Detection of tibial quality parameters

The stiffness and Young’s modulus of the tibia were not significantly different between the TD and NTD chickens (*p* > 0.05). BMC, BMD, bone ash content, calcium content, and phosphorus content values were lower in TD birds than in the NTD broilers (*p* < 0.05) (Table 1).

**TABLE 1:** Changes in bone quality parameters of TD broilers.

Items	NTD	TD	*p*-value
Stiffness, kN	120.50 ± 10.17	124.38 ± 11.04	0.59
BMC, g	7.74 ± 0.41	6.78 ± 0.98	0.01
BMD, g/cm^2^	0.34 ± 0.01	0.27 ± 0.04	3.00e-5
YM, Gpa	3.57 ± 0.40	3.73 ± 0.6	0.55
Bone ash content,%	46.17 ± 0.8	42.64 ± 0.58	1.90e-11
Calcium content,%	17.24 ± 0.54	15.35 ± 0.65	9.27E-08
Phosphorus content%	8.35 ± 0.17	7.41 ± 0.16	2.35E-12

All values are shown as mean ± standard deviation, n = 12. BMC, bone mineral content; BMD, bone mineral density; YM, Young’s modulus.

### 3.3 Transcriptome analysis of growth plates

**FIGURE 2:**
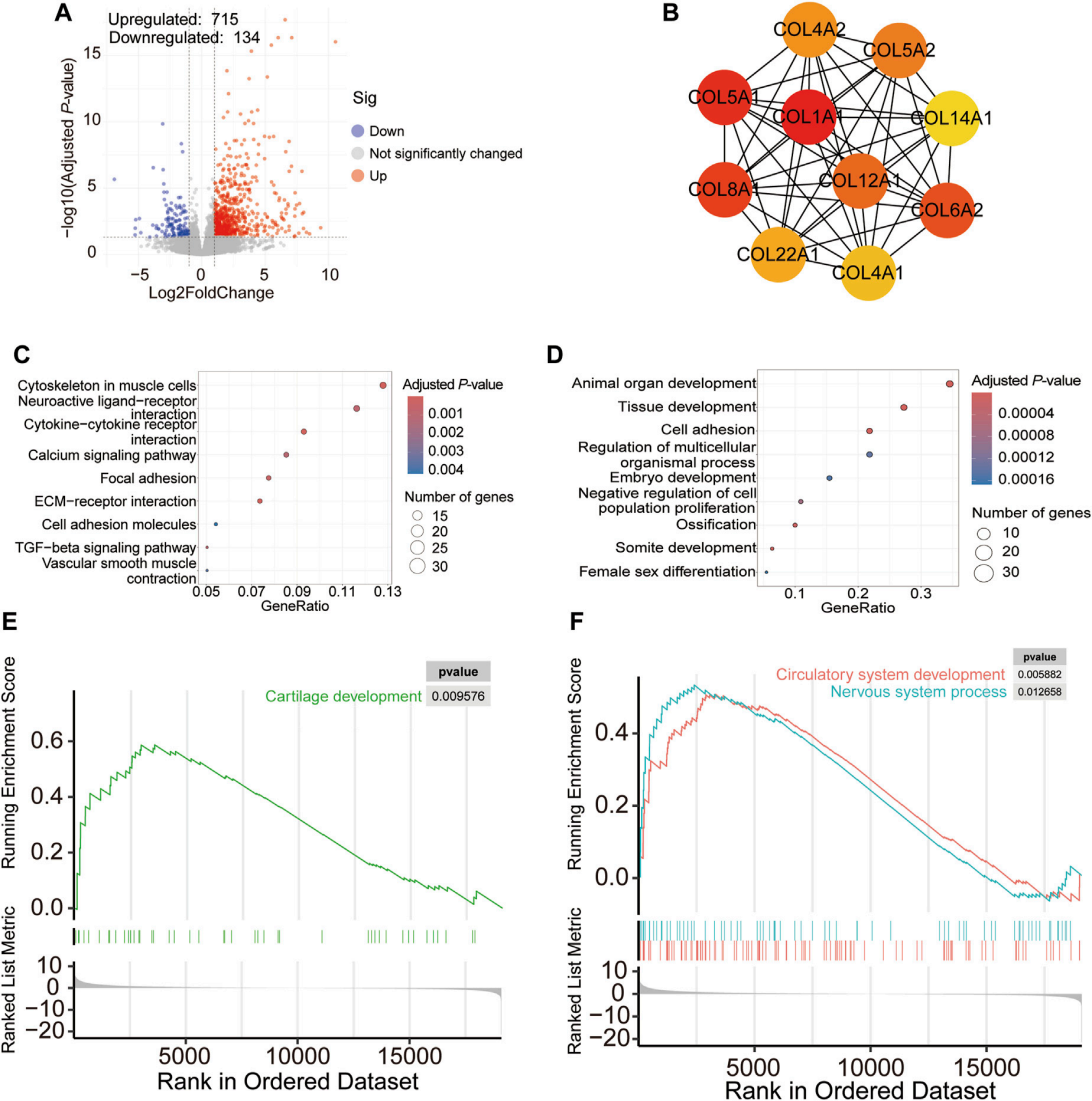
Volcano plot showing differentially expressed genes in growth plates between broilers with TD and NTD chickens **(A)**; protein-protein interaction network of the top 10 DEGs **(B)**; KEGG **(C)** and GO **(D)** analyses of DEGs in broilers with TD and NTD; GSEA analysis revealed upregulated pathways in bone development **(E)** and system development **(F)**.

Differential analysis revealed that the expressions of 715 genes were significantly upregulated in chickens suffering from TD, whereas those of 134 genes were downregulated (Figure 2A). Based on the protein-protein interaction network, *COL1A1*, *COL5A1*, and *COL8A1* were considered to be essential genes that may cause TD (Figure 2B). KEGG analysis showed that the DEGs were involved in the ECM-receptor interaction, cytokine-cytokine receptor interaction, focal adhesion, calcium signaling pathway, and TGF-beta signaling pathway (Figure 2C). GO analysis revealed that the biological processes involved in animal organ development, cell adhesion, and ossification were enriched (Figure 2D).

We used GSEA for further investigation to gain a comprehensive understanding of the changes in gene expression between TD and NTD birds. The results showed that the signaling pathways involved in cartilage, circulatory, and nervous system development were upregulated in broilers with TD (Figures 2E, F). These results suggest that the circulatory and nervous systems play a role in TD.

### 3.4 Transcriptome analysis of blood

**FIGURE 3:**
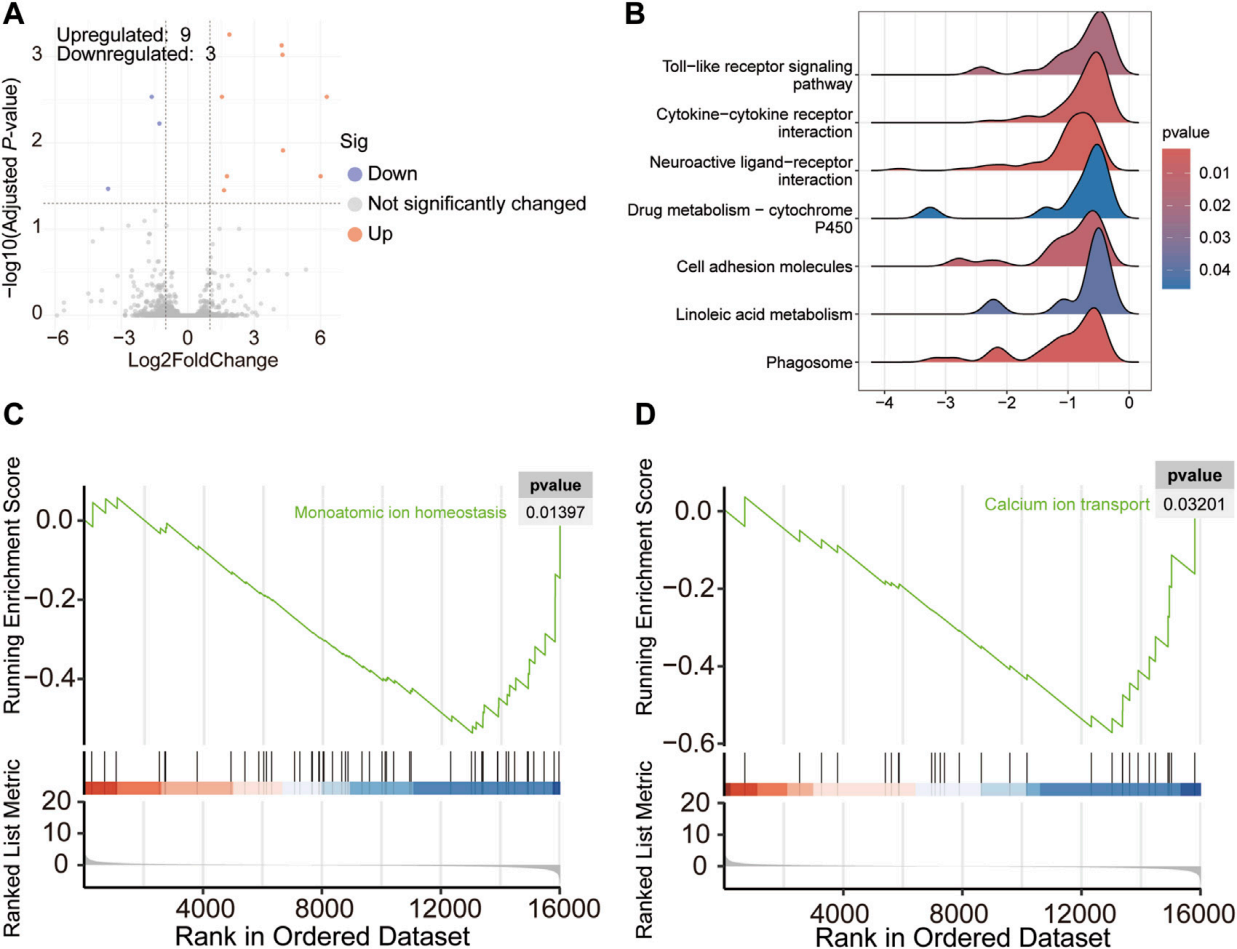
Volcano plot showing differentially expressed genes in the blood of broiler chickens with TD and NTD **(A)**; Seven significantly decreased signaling pathways based on KEGG analysis of GSEA analysis in broiler chickens with TD and NTD **(B)**; GO analysis of GSEA detected downregulated pathways **(C,D)**.

Differential analysis revealed that the expressions of nine genes were upregulated and those of three were downregulated in chickens with TD (Figure 3A). In addition, GSEA analysis showed that the altered genes were involved in signaling pathways, such as phagosome, linoleic acid metabolism, cell adhesion molecule, cytokine-cytokine receptor interaction, and neuroactive ligand-receptor interaction, which were found to be downregulated, as shown in Figure 3B. In addition, according to the GO analysis, the signaling pathways related to the homeostasis of monatomic ions and calcium ion transport were reduced in the blood of broiler chickens with TD (Figures 3C, D).

## 4 Discussion

Bone development is a complex process influenced by various physiological and biochemical factors, as well as by the proliferation and differentiation of chondrocytes in the growth plate and vascular development. TD affects broiler chickens and is caused by the accumulation of avascular and non-mineralized chondrocytes on the growth plate. This condition can lead to limited mobility and standing ability, feeding difficulties, and developmental deformities of the tibia (Guitart et al., 1996; Genin et al., 2012). TD is responsible for 30% of bone diseases in birds, with an incidence of approximately 10%, leading to significant losses in the poultry industry (Li et al., 2008; Mehmood et al., 2017). However, the mechanism of TD is not well understood. Most studies on broiler chickens with TD have been based on a thiram-induced model, which may differ from the natural occurrence of TD in individuals. Therefore, we conducted a study on a yellow-feather broiler population for 10 weeks to investigate the morbidity and bone quality parameters of TD birds and to explore the regulatory mechanisms of TD using transcriptome technology.

In this study, we used a digital X-ray machine to investigate TD phenotypes. Our results showed that cartilage at the metaphysis of the tibia was irregular in TD broiler chickens, resulting in the formation of white jade cartilage plugs in the growth plate. The incidence rate of TD was the highest at 6 weeks and decreased to 12% by week 10, which may be due to the self-healing properties of TD broilers. TD broiler chickens also showed a significant decrease in body weight at 6, 8, and 10 weeks of age, possibly due to anorexia. The length, diameter, and density of the tibia were also measured. The length of the tibia showed no changes in the TD chickens after 6 weeks but decreased significantly after 8 and 10 weeks. There was no difference in the diameter of the tibia between the TD and NTD chickens. The density of the tibia of TD broiler chickens was significantly reduced compared to that of NTD broilers, which is consistent with a previous study (Jiang et al., 2019). The tibia of TD broiler chickens typically exhibits avascularity at the metaphysis, which may lead to an inadequate supply of the nutrients and minerals required for osteogenesis. This ultimately leads to changes in the morphological parameters of the tibia in the TD broilers. To assess bone quality parameters in chickens, stiffness, BMC, BMD, YM, bone ash content, and calcium and phosphorus content are important indicators that are frequently used (Newman and Leeson, 1998; Regmi et al., 2016; Sharma et al., 2021). No significant differences in tibial stiffness and YM were observed between the TD and NTD groups. However, lower BMC, BMD, bone ash content, calcium content, and phosphorus content of the tibia were observed in the TD group. Previous studies have reported that regular exercise is beneficial for bone development and that broiler chickens raised on the floor had higher stiffness, BMC, BMD, and YM levels than those raised in cages (Regmi et al., 2016; Sharma et al., 2021). The reduced tibial quality parameters in TD chickens may be due to the reduced mobility and avascular condition of the tibial metaphysis. Calcium and phosphorus are essential for tibial development. Studies have shown that broiler chickens with a high incidence of TD have higher plasma calcium and phosphorus concentrations than those with a low incidence of TD (Yalçin et al., 2000).

Transcriptome analysis revealed 849 DEGs in the growth plate between TD and NTD chickens. The protein-protein interaction network indicated that genes encoding the alpha chain of type XII collagen (for example, *COL1A1*, *COL5A1*, and *COL8A1*) are crucial, and their abundance may have caused TD. KEGG analysis showed that these DEGs from the growth plates were involved in ECM-receptor interaction, cytokine-cytokine receptor interaction, focal adhesion, calcium signaling, and TGF-β signaling pathways. In addition, GO-enriched analysis revealed that growth plate DEGs regulate animal organ development, cell adhesion, and ossification. Mutation of COL1A1 results in disruption of the collagen I triple helix and causes inherited brittle bone disease osteogenesis imperfecta (Blank et al., 2022). In one study, it was discovered that miR-92a-1-5p was found to target COL1A1, which promotes osteoclast differentiation and inhibits osteoblastogenesis (Yu et al., 2021). Another study found that ECM-receptor interaction, cytokine-cytokine receptor interaction, focal adhesion, and TGF-β signaling pathways were enriched in the growth plate in a thiram-induced TD model, which is consistent with the results of our study (Mo et al., 2023). In addition, GSEA also identified the signaling pathways involved in cartilage development, circulatory system development, and nervous system development. Hyperglycemia was considered a pathogenic factor for TD in a previous study (Mo et al., 2023); therefore, the blood transcriptome was examined. The results showed that nine and three genes were identified with upregulated and downregulated expression, respectively, in TD birds. GSEA analysis revealed that cytokine-cytokine receptor interaction, toll-like receptor signaling, and neuroactive ligand-receptor interaction were significantly altered, consistent with the transcriptome results of the growth plate in TD chickens. Linoleic acid metabolism was decreased in the blood of TD birds, which could regulate the RANKL/RANK/OPG signaling pathway and prevent bone loss, and may also affect the occurrence of TD (Abdulrahman et al., 2023). In addition, monoatomic ion homeostasis and calcium ion transport were reduced in the blood of the TD group mice. In the results of tibia quality parameters, tibia calcium and phosphorus content were found to be significantly decreased in TD broiler chickens. High serum calcium and phosphorus levels have been reported previously. It has been hypothesized that impaired calcium and phosphorus uptake due to avascularity of the tibial metaphysis contributes to changed calcium and phosphorus levels in the tibia and serum (Yalçin et al., 2000).

## 5 Conclusion

In a recent study, we found that TD morbidity rate of the yellow-feathered chickens was 22% at 6 weeks. TD leads to reduced growth in chickens and affects various bone-related indices such as BMC, BMD, bone ash, calcium, and phosphorus in the tibia. The transcriptome results from tibial growth plates and blood suggest that ion homeostasis and transport of the circulatory system, together with different signaling pathways in the growth plate, may contribute to the development of TD in broiler chickens. This study provides new insights into the development of TD in chickens.

## Data Availability

The datasets presented in this study can be found in online repositories. The names of the repository/repositories and accession number(s) can be found below: https://ngdc.cncb.ac.cn/gsub/submit/gsa/subCRA025975/finishedOverview, CRA016204.
